# ‘It's Got Its Ups and Downs’: What People With Intellectual Disabilities Living in Supported Living and Residential Care Like and Dislike About Their Home

**DOI:** 10.1111/jar.13313

**Published:** 2024-10-24

**Authors:** Francesca Ribenfors, Lauren Blood, Chris Hatton, Anna Marriott

**Affiliations:** ^1^ Manchester Metropolitan University Manchester UK; ^2^ National Development Team for Inclusion Bath UK

**Keywords:** belonging, housing, intellectual disability, residential care, supported living

## Abstract

**Background:**

Given the current sociopolitical climate, people with intellectual disabilities are spending more time at home. Much housing‐related research focuses on informant‐completed measures and quantifiable outcomes. By contrast, this article explores the perspectives of adults with intellectual disabilities concerning what they liked or disliked about their homes.

**Method:**

Data is drawn from 53 semi‐structured interviews with people with intellectual disabilities in supported living or residential care in England.

**Results:**

Three themes were generated: space and place; people make or break a home; and day‐to‐day autonomy. These highlight the importance of belonging and the significance of other people in the creation of ‘home’.

**Conclusion:**

If people are to flourish, attention must be paid to aspects of the home that provide comfort, enjoyment, and a sense of belonging. These findings can benefit professionals, family members and people with intellectual disabilities, when considering current or future living arrangements.

## 1 Background

Where we live, including the building, environment, and location, affects our physical, social and mental wellbeing (Veitch 2008; Andrews et al. 2012). When a house is a home, it offers the boundary and space that supports our need to belong (Blunt and Dowling 2006; Marshall 2008), acts as a place of safety or refuge, and reflects our culture and identity (Després 1991; Marshall 2008). However, a house is not automatically a home. Instead, homes are a personal experience. They are created but ever‐changing, affected by the power relations that flow in and around the home and the material interactions of everyday life (Blunt and Dowling 2006).

Power relations affecting experiences of home are particularly salient for people with intellectual disabilities due to a long history of institutionalisation and deprivation of autonomy. Over recent decades, there has been a policy shift from larger residential institutions to smaller community‐based homes, supported living and other flexible arrangements (Mansell and Beadle‐Brown 2010). However, despite this, people with intellectual disabilities continue to live in institutionalised settings. Williams et al. (2023), reported on the prolonged stays people with intellectual disabilities face in assessment and treatment units despite no clinical need, while ongoing campaigns such as ‘Homes not Hospitals’ (BASW 2024) and ‘Stolen Lives’ (Cavanagh and Hinksman 2024) emphasise how the heavy restrictions within these settings deprive people of a home of their own.

Several reviews demonstrate better outcomes for people in individualised community settings compared to larger congregate settings (e.g., Kozma, Mansell, and Beadle‐Brown 2009; Chowdry and Benson 2011; McCarron et al. 2019; Oliver et al. 2022). Supported living is intended to enhance quality of life outcomes and enable people to live in ‘real homes of their own giving people more control over who supports them, where they live, whom they live with, and the lifestyle they wish to lead (Kinsella 1993, 6)’. Within supported living the person owns or rents their home with a tenancy agreement. Accommodation and support should be provided by separate agencies and the home itself is not regulated. This stands in contrast to residential care where people are provided with a room in a home with meals, care and support all include and, within the UK, the home is regulated by the Care Quality Commission (CQC). Residents typically have little choice in the organisation or running of the home (e.g., who supports them or who they live with) and the number of residents is often greater than in supported living, although more than six residents is highly discouraged by the CQC (Harflett et al. 2017). While supported living has afforded some people more autonomy over their day‐to‐day lives (Bigby, Bould, and Beadle‐Brown 2017), some research suggests that people with intellectual disabilities continue to have little choice over where and with whom they live (Stancliffe et al. 2011; Salmon et al. 2019; Blood et al. 2023).

Less attention has been paid to the architectural design of homes for people with intellectual disabilities (Salmon et al. 2019). However, unsuitable surroundings can cause distress, accidents, and reduced independence (Bradley and Korossy 2016; Nagib and Williams 2017; Casson et al. 2021). Reviewing theories and models of home design, Yong, Haines, and Joseph (2023) concluded home environments for people with intellectual disabilities should offer safety, comfort, control, choice, skill acquisition and support person–environment interaction. This may be challenging for supported living homes compared to purposefully designed residential homes as they are typically ordinary houses that are not designed for shared living (Clark et al. 2018) or disabled people (Imrie 2004).

Confirming the importance of the physical environment, participants in Salmon et al.'s (2019) study prioritised location, accessible design and space for guests when discussing living arrangements. This research is notable for eliciting perspectives of people with intellectual disabilities through an inclusive methodology with people with intellectual disabilities on the research team. Similarly focusing on the opinions of people with intellectual disabilities, McConkey et al. (2004), used focus groups with 180 participants to explore their thoughts on living arrangements. Participants living in their family home and in residential settings valued contact with family and friends, access to local facilities, participation in household tasks and having a personalised bedroom. Although participants were reluctant to discuss areas of their home they disliked, unpleasant neighbours and antisocial behaviour were mentioned by people living independently, with family or in supported living. Similar issues were reported by participants living outside of registered settings in Blood et al. (2023) and are likely related to links between poverty, disability and living in socially disadvantaged areas (McConkey et al. 2004; Blood et al. 2023). Another study focusing on the perspectives of 10 participants with intellectual disabilities, aged 18–23, living in their family home regarding their current and future living arrangements (Cahill and Guerin 2023), found similar themes to McConkey et al. (2004) relating to personalised space and familiar community.

These papers stand in contrast to much housing‐related research concerning people with intellectual disabilities, where observational methods and informant‐completed objective and quantifiable measures dominate and there continues to be a need for qualitative housing‐related research that foregrounds the perspectives of people with intellectual disabilities (Bigby, Bould, and Beadle‐Brown 2017). This is particularly pertinent in the current sociopolitical climate with disabled people spending more time at home (Malli et al. 2018). During the COVID‐19 pandemic, many people with intellectual disabilities had to ‘shield’ within their homes (Taggart et al. 2022) whilst reduced support and social care services contributed to social isolation (Flynn et al. 2021; Scherer et al. 2023). These challenges added to pre‐existing austerity‐driven reductions in the quality and provision of services which had already rendered some people housebound and others isolated (Malli et al. 2018). A report by Mencap (2012), for example, found 25% of people with an intellectual disability surveyed spent less than 1 h a day outside their home.

Therefore, in the wake of the pandemic and with recognition of the time people with intellectual disabilities spend at home, this paper addresses subjective experiences, exploring what people with intellectual disabilities living in residential care or supported living like or dislike about where they live. While the findings are intended to inform areas such as support and commissioning, we wish to emphasise that responding to individual preferences to create the desired home is not a substitute for supporting people to lead fulfilling lives outside of the home. Rather, both are necessary for people to flourish.

## 2 Methods

**FIGURE 1:**
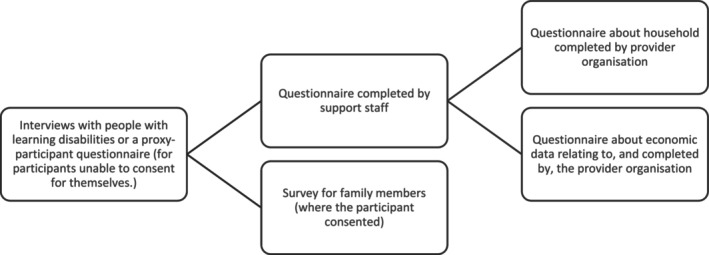
Summary of research methods included in the wider study.

This paper draws on data from a mixed‐method study, 200 Lives: Evaluating supported living and residential care for adults with learning disabilities, funded by the National Institute for Health and Care Research. Ethical approval was received from the Social Care Research Ethics Committee (ref 20/IEC08/0041). Coinciding with the COVID‐19 pandemic, data was collected between March and December 2021 using surveys and interviews with people with intellectual disabilities, proxy‐participants, support staff, organisations providing residential care or supported living, and family members as shown in Figure 1. This paper focuses solely on data arising from the interviews with people with intellectual disabilities and the proxy‐participant questionnaires used following a consultee process for participants who were unable to consent to participation (Dobson 2008). Other aspects of the research are reported elsewhere (Hatton et al., 2022).

### 2.1 Recruitment

The project was advertised on social media and disseminated through the research team's networks. Residential care home providers and organisations supporting people in supported living shared an easy‐read information booklet and a YouTube video about the project with eligible people. Details of people interested were then passed on to the research team who made contact to discuss the research, explain the consent process and arrange an interview with formal informed consent gained prior to the interview commencing. Following Dobson (2008) and the 2005 Mental Capacity Act, a consultee process was enacted to include people who could not consent for themselves. Eligible participants were people with an intellectual disability in supported living or residential care aged between 18 and 74 years old and who had lived in their current home for at least 6 months. The upper age limit was initially 64 to focus on working age adults with intellectual disabilities who were not in services for older people, where very different sets of organisational procedures (and costs) apply. During the project, it became clear that participating supported living and residential care services included some people aged 65 or over, so to include more participants we expanded the upper age limit to 74. Very few supported living and residential care services without nursing care supported people beyond this age.

### 2.2 Participants

One hundred and seven people with intellectual disabilities participated, 14 of whom were included via the consultee process. Seventy‐seven participants lived in supported living and 30 participants lived in residential care. Participants were spread across 16 organisations with 1–19 participants per organisation.

### 2.3 Data Collection

Semi‐structured interviews were conducted with participants over video call, phone or face‐to‐face during a home visit. Where possible people were given a choice of how they participated. However, due to national lockdowns and additional restrictions imposed by providers, most interviews took place via video call. The interview schedule included open‐ended and closed questions to generate both quantitative and qualitative data. Of relevance to this paper, the schedule included questions about what participants liked or disliked about their home and if there was anything about it they would change. The schedule was developed with input from the project's advisory group (comprising people with intellectual disabilities, family members and support providers), public engagement events with people with intellectual disabilities and support staff, and a review of previous research. Researchers were responsive to the individual needs of participants and adapted the number and wording of questions, and topics covered within interviews to suit the individual. Likewise, interviews took place over one to three session/s depending on individual preference, and participants could choose whether they had someone to support them during the interviews. With consent, interviews were audio‐recorded. Where the consultee process had been followed, a staff member answered the interview questions via a ‘Proxy‐Participant’ questionnaire.

### 2.4 Analysis

Due to the number of participants, although the quantitative data from all interviews was analysed and reported elsewhere (Hatton et al., 2022), a sub‐group of interviews was selected via purposive sampling for analysis of the open‐ended questions. Following discussion amongst the research team to ensure that a range of participants and housing set‐ups were represented, 26 interviews with people in residential care (including 7 people who participated via a proxy‐questionnaire) and 27 interviews with people in supported living (including 2 people who participated via a proxy‐questionnaire) were selected. Table 1 shows an overview of the participants included within this analysis.

**TABLE 1:** Overview of participants included in the analysis.

Age (years)	Mean Range	42.6 (13.56) 19–72
Gender	Men Women Other	57.4% 37.0% 1.85%
Ethnicity	White (all groups) Asian/Asian British Black/Black British Mixed heritage	83.3% 1.9% 5.6% 3.7%
Housing model	Supported living Residential care	27 26

Responses to the open‐ended questions were transcribed verbatim and anonymised by two research team members. The researchers created a framework matrix (Gale et al. 2013) to collate and code data extracts relevant to the research questions. Coding was an iterative process and involved moving back and forth between the data, the codes, the transcripts, and researcher discussions. Although an inductive approach to coding was used (Braun and Clarke 2012), part of the process was deductive as the data was broadly organised into topics via the matrix. Once all extracts were coded, the codes were grouped into themes following further discussion amongst the research team.

## 3 Results

Relating to what people like or dislike about their home, the analysis generated three themes: (1) Space and Place, (2) People Make and Break a Home, and (3) Day‐to‐Day Autonomy. Space and Place contained three subthemes: (1.1) Claiming Space, (1.2) Location, Location, Location and (1.3) Problems with the Physical Environment.

### 3.1 Space and Place

#### 3.1.1 Claiming Space

Participants, particularly those living with others across housing types, liked claiming space within their home, carving out areas to personalise and make their own. For some, this meant shutting their bedroom door, and other people knocking before entering, deciding how their bedroom was decorated, or choosing and purchasing new furniture. However, claimed spaces extended beyond individual bedrooms into other areas of the property, as participants spoke about having their own shed, personalising and maintaining the garden, using the garage, or as in the case of one participant, having a spot within the kitchen as the staff member explained:

He has his own spots around the house, specifically in the kitchen he has his own island which he has all of his pens and paper and a radio he listens to his music on. (Proxy participant 21, RC)

For some people the claiming of space was less tangible but rather a sense of ownership and control was evident in knowing where everything belonged, and the pride and enjoyment taken from keeping a clean and tidy home:

I love it there. I can do my own things like…washing my own washing, hoovering, stripping my bed, cooking my own meals. You can decide what you want to do. (P2, SL)

He gets unsettled if he can't find [his things] or if someone moves them. He likes the house to be kept clean and staff to respect the house. He asks them to mop the floor every night. (Proxy Participant 13, SL)

The claimed spaces served multiple purposes. First, they fostered a sense of control and belonging over the environment, second, they enabled the pursuit of hobbies or interests, and, thirdly, in some instances, they provided a place of sanctuary. For example, a garden shed provided one participant with space for his model railway but was also somewhere he escaped a difficult housemate: ‘I go out to my shed and have a cigarette out there to get out the way’ (P31, RC). Someone else described retreating to their bedroom to avoid housemates and another participant found a sense of safety by putting everything away correctly at the end of the day.

#### 3.1.2 Location, Location, Location

The home's location was important to participants in both supported living and residential care. People liked living in quiet locations as one person commented: ‘The area is quiet where we are and there's not much trouble here’ (P25, RC).

Participants also appreciated living near to where they grew up and enjoyed seeing family, friends, or former neighbours when out and about. This was important even when participants were no longer close to their family. For one person who was not in regular contact with his family, he appreciated occasionally bumping into them and saying hello:

I just bump into them…it feels quite good if I haven't seen them in a long time, it is nice to see them again. (P31, RC)

Participants in both types of housing liked being able to walk or use public transport to get places without staff support. This fostered their sense of independence and freedom and contributed to a fulfilling life through participation in the community for example through church, football or work.

I think the shops, walking along the canal into town. ASDA obviously… I feel a bit more freedom here. (P7, SL)

I go out every day and hardly spend any time at home…sometimes I go over to the main house or sometimes I am just out at work, or I am out with my friends, so I have a very busy life. (P37, RC)

Participants in supported living valued good relationships with their neighbours. For example, one proxy‐participant explained how the person they supported in supported living liked street parties and the neighbours returning their balls when they went over the fence. Another participant described how their neighbour sometimes brought round meals for them and another stated: ‘I enjoy…making sure everyone else is OK like neighbours and friends and stuff’ (P29, SL).

Conversely the importance of the neighbourhood could work both ways, as for some participants with minimal support in supported living, their location contributed to feeling unsafe within their home due to encountering difficult neighbours and antisocial behaviour:

I don't like it because of the area. We have a neighbour the other side…she likes to take the biscuit and take the piss out of people, and I just want out…I don't like the area as my partner has been mugged so many times; six times by the same person. (P36, SL)

People throw glass. It is a violent place at times and the police come out. The front door was broken, and it took a while to fix, and things keep going wrong. I just want to move to a bungalow with a shed for my bike—I have to bring it upstairs at the moment… it is not a good area to live. (P30, SL)

#### 3.1.3 Problems With the Physical Environment

Some participants were unhappy with physical aspects of their home and problems relating to maintenance, accessibility and the property's size. Some accessibility issues were raised by people living in residential homes, for example, one person was unable to access the laundry facilities in their residential home which was affecting their independence:

I used to go to college, and they had a laundry so I could do my washing and ironing, folding my clothes and drying. But here there is a small laundry, and it won't fit my chair so I can't even put clothes in the laundry. (P24, RC)

However, most people reporting on physical aspects were in supported living and lived alone or with fewer housemates.

Issues with property size could be exacerbated when staff were also present as ‘it can get quite crowded’ (P7, SL) and participants were unhappy when maintenance issues, such as issues with hot water and heating, were not addressed. Often this was attributed to problematic landlords:

Landlord is horrible, he is arrogant, he doesn't come and do any alterations to the house, he puts the rent up. (P23, SL)

Similarly, participants were frustrated when landlords would not make desired adaptations such as a built‐in shower seat or a wet room and one staff member believed that these issues may be resolved more efficiently in a residential home:

He needs a specialist environment and trying to get that with the set‐up can prove difficult. I think in a registered service it would just be done and it would be perfect for him… there are massive triggers for him everywhere in that house…The lights are a constant source of triggering his behaviours… So, I've been battling with the [housing] providers just to change the lighting… (Proxy‐participant 13, SL)

### 3.2 People Make and Break a Home

Relationships with other people within the house were important to participants across settings. Some focused on living with friends, celebrating events together and enjoying their housemates' company. For example, one participant stated: ‘It's absolutely brilliant, we have a laugh here…We all get along’ (P4, SL) whilst another liked his home because of ‘the people I live with’ (P25, RC). However, for other participants, particularly people with higher support needs or living in residential homes, staff were the focus. Participants drew attention to the helpfulness of staff when describing what they liked about their home:

They are very nice and look after me to the best of their ability and whenever I need anything like shopping fetching for a certain date and so forth, they are very prompt at that. (P26, RC)

All the staff where we live are good, they help you through things…I like talking to staff to sit and talk about how I feel. (P5, RC)

Additionally, some participants liked their current home because they were treated better than in previous situations:

The staff treat you with more respect…I love it, it's a lot better place. (P4, SL)

People listen to me here. (P28, RC)

However, issues relating to staff were also raised; a lack of staff affected what people could do, some staff treated the house as a workplace rather than a home, and, as for the following participants, some staff were rude or unhelpful:

I just didn't like the way how she was being with me, she was snapping at me and speaking with me out of order, and at the end of the day I just don't think that is right as I don't live here to be upset, I live here to be happy. (P26, RC)

I asked him nicely, but he said do it yourself…he didn't help me. I don't want him anymore. (P33, RC)

The transience of staff and housemates also affected participants' enjoyment of their home:

I don't like people leaving and I don't like change. (P8, RC)

It's all gone downhill at the moment. Every night I just think about what could have happened if she was there. (P9, RC)

Additional problems with housemates were encountered in both types of living situation. As demonstrated in the extracts below, these issues were often related to the noise and behaviour of housemates, or a mismatch in support needs and the subsequent frustration this could cause. Sometimes participants felt unsafe:

Sometimes it gets a little bit hectic because there are ten people doing ten different things at the same time…It does get a bit frustrating sometimes…like other people I live with can't talk back to me…or the ones that you can have a conversation with, they forget what you said so…it's like ‘shut up I am trying to watch tv’ and then two hours later you are telling them to shut up again because I am trying to watch tv. But other than that, I like living here but sometimes I wish I could get away at the same time but at the minute you can't. (P24, RC)

My house is alright but there are some people I have disagreements with…the lad that I was on about tried to threaten me with a knife. (P32, SL)

Coping strategies for managing these difficulties were described. These included using headphones to block out noise in communal areas, avoiding trips on the minibus with certain housemates, and seeking out quiet, private spaces.

Participants typically believed that who they lived with, or the lack of support experienced, was outside their control. Therefore, even though they disliked the situation they felt they just had to accept it:

They can't actually do anything about it as such… sometimes I am like ‘please do something about this’ but then they can't so it is the way it is. Sometimes you have to put up with people you don't like sometimes. (P24, RC)

Sometimes it can pop up that there are not enough staff to go out, so I just go back to doing my embroidery, and word searches and watching the quizzes and sport on tele. (P32, SL)

### 3.3 Day‐to‐Day Autonomy

Participants liked being in charge of their daily lives and choosing what and when they did things. For some people this involved going out when they wanted (either alone or with support), whilst for others it included watching what they wanted on TV, choosing to lounge on their sofa or cooking for themselves. This control was often associated with freedom:

My freedom…. getting out, I don't have to be back at a certain time. I can do what I want… can take charge of my life, got my own front key. (P3, SL)

Again, comparisons were often made to previous more restrictive living situations. For example, the participant quoted above (P3, SL) also commented ‘I've got my independence back’ as they had moved from a shared house of 12 into their own flat. Another participant who had moved from a long‐term hospital into supported living stated: ‘it's much better, more freer, I mean I have been doing more things’ (P23, SL) and a third stated:

Freedom…I can go out when I want and come in when I want and watch what I want to see without being told what not to watch and stuff and people coming and saying oh no it's my turn to watch it now…I can cook when I want and have it how I want…Like when I was in a children's home and foster care and hostel, like with nine other men so we had to share everything. I had my own bedroom but that was about it. (P22, SL)

However, the autonomy experienced was often facilitated by, or contingent on, others. Participants valued being treated as adults, having decisions respected, and staff supporting them to go out when they wanted to as in the following example:

They accommodate the things I want to do like work. If I need to go out, they allocate me somebody. Say I have been allocated a job they would allocate me a driver. (P24, RC)

## 4 Discussion

When participants in our study discussed what they liked or disliked about where they lived, it was the physical space and location, the people within and around the house, and the ability to have control over their everyday life that mattered; themes similar to those highlighted in previous research (McConkey et al. 2004; Cahill and Guerin 2023). These findings and aspects that matter to individuals with intellectual disabilities should be considered when planning or quality‐checking homes. This is necessary as differences can emerge between the views of people with intellectual disabilities and authoritative figures around them (McConkey et al. 2004). Additionally, the findings indicate that when listening to people with intellectual disabilities discussing their home, it is important to reflect on their past living situations. Comparisons are often made to previous more restrictive living environments which may provide a limited frame of reference and set a low bar for what is considered acceptable (Blood et al. 2023).

Aspects of their living environment that participants liked also align with notions of home whereby it is ‘the space itself and the people around and within it’ (Murray 2018, 287) that make a home rather than technicalities such as tenancy status. Indeed, looking at the findings in conjunction with literature on ‘homeliness’ reveals how, for participants in both types of setting, the favoured aspects supported feelings of belonging and connection.

### 4.1 Belonging Within the Home

Participants valued having spaces within the home that they could claim as their own. Whilst these spaces often provided room for hobbies or interests, they also created an opportunity for the home to become ‘a place to which you belong, and which belongs to you’ (Murray 2018, 287). Acting upon and modifying one's environment, for example through decoration or choice and placement of furniture, is one way in which a house can become a home (Després 1991). Doing so responds to a need to ‘claim and defend one's territory’ (Yong, Haines, and Joseph 2023, 263) which is commonly achieved by marking out space as one's own (Yong, Haines, and Joseph 2023, 263). For people with intellectual disabilities in shared homes the bedroom often provides this personalised space (Murray 2018; McConkey et al. 2004; Cahill and Guerin 2023). However, our findings highlight how spaces beyond the bedroom are also important with garages, gardens, sheds and other spots around the house providing spaces that people could claim, make their own and use as they wished.

The spaces carved out supported people to feel safe when living with difficult housemates. Whilst we advocate that people are not placed in this position in the first place, private spaces can act as an interim coping strategy. In line with this, Yong, Haines, and Joseph (2023) highlighted the importance of people within shared homes having spaces of refuge to avoid others as needed. Although most people will have a bedroom that can provide a safe space, not everyone will feel safe within this space. Furthermore, there is a danger people may become confined to their bedroom, which may reduce their sense of ownership or belonging over the remaining house. Therefore, having additional spaces within and around the home that can act as a place of refuge and provide people with opportunities to escape or avoid difficult situations whilst being an enjoyable place to spend time may help foster feelings of belonging and connection to the home. This is especially important for people whose sense of home may be compromised by the very nature of living with people they do not wish to or do not get along with (with the impact others can have on the creation of home discussed in the next section). Likewise, as feelings of home extend beyond the immediate building (Blunt and Dowling 2006) belonging can be strengthened when the home is in a familiar location close to family or friends, or alongside good neighbours, as appreciated by participants in this study. Conversely, feeling uneasy in one's home or the local area due to difficult neighbours or antisocial behaviour disrupts belonging and leads to people wanting to move (Blood et al. 2023).

Our findings indicate that maintenance of the space is also important as participants raised maintenance, accessibility and adaptation issues when discussing what they disliked about their home. This may be of particular concern when people experience sensory sensitivities due to the building design (Foundations 2021). Although these issues pose practical challenges, when aspects of the home that require fixing or changing remain undone it disrupts one's sense of belonging (Murray 2018). A house where you cannot move freely, or that is not adapted for your needs is less likely to feel homely or welcoming (Imrie 2004; Blunt and Dowling 2006).

Imrie (2004, 760) suggests impairment poses a challenge to ideal conceptions of the home as a place of privacy, sanctuary, and security, and reinforces the notion that these aspects of home are ‘always conditional, contingent, never secure’. However, in keeping with the social model of disability, based on our findings we suggest it is not impairment that is challenging, but rather that required adaptations are not made or considered from the outset, something landlords, support staff, and providers could play a role in rectifying.

### 4.2 People Within and Around the Home

Indeed, how other people affected participants' experiences of their home threads throughout the findings. Similar to Imrie's (2004) assertion above, requiring support within the home challenges the privacy of the home as support staff and health professionals enter the home often in positions of authority (Dyck et al. 2005). Endeavours to ensure people have their own front door key and control entry to the home, as visible within the Real Tenancy Test (NDTi 2015) and Reach Standards (Warren and Giles 2019) for supported living, help mitigate the tension between public and private by reiterating the privacy of the home. Nevertheless, as people with intellectual disabilities continue to have limited choice over housemates and staff (Stancliffe et al. 2011; Tichá et al. 2012; Salmon et al. 2019; Blood et al. 2023), the navigation of difficult relationships poses an additional hurdle in the creation and preservation of home. Participants evoking their relationships with support staff when discussing what they liked about their home suggests that staff were not peripheral to the home, rather a fundamental part of it.

As in Bigby, Bould, and Beadle‐Brown (2017) participants drew attention to characteristics of staff they appreciated, whilst others highlighted instances when unhelpfulness or disrespect shown by staff affected their enjoyment of their present or previous home. The prominence of staff was also evident when participants discussed autonomy over their day‐to‐day life. Control over aspects of daily life such as how to spend their time is more readily available to people with intellectual disabilities than support‐related choices (i.e., choices over housemates and support staff and where to live) (Tichá et al. 2012). However, it is apparent within the findings that even these everyday choices remain largely contingent on staff respecting, facilitating, or ‘accommodating’ participants' decisions. This places people with intellectual disabilities in a precarious position, for whilst autonomy can be enabled it can also be denied.

A similar precarity emerges in the transience of staff and housemates that participants discussed. At any point, favoured support staff or housemates can leave, upsetting the stability and enjoyment of the home. This also adds meaning to the notion that homes are never static but rather ‘ever changing and differently experienced by the individuals who weave and flow their paths through and around these places’ (Murray 2018, 17). Whilst there is no clear solution to these difficulties, supporting people to maintain contact with staff and housemates who move on may help manage some of the difficulties experienced through the disruption of stability. Salmon et al. (2019) suggested involving people with intellectual disabilities in recruitment may help set the tone for subsequent relationships and ensure individual preferences are considered. Additionally, paying attention to the home's location may enhance a person's autonomy. As for participants within this study, being within walking distance of local amenities or on public transport routes can positively affect one's life (Mansell et al. 1987; Andrews et al. 2012) and encourage a sense of freedom and independence that is not predicated on the will or ability of others.

### 4.3 Limitations

Whilst we endeavoured to be inclusive, involving people unable to consent to participation via the consultee process and a ‘proxy‐participant’ questionnaire was a decision of pragmatism due to the large sample size and the small research team. Nevertheless, this approach is problematic (Nind 2008) and more creative methods may better include people with profound and multiple intellectual disabilities. Related to the methodological approach, challenges can arise when asking people with intellectual disabilities to report on aspects of their home they dislike (McConkey et al. 2004; McGlaughlin and Gorfin 2004). Therefore, we recommend our findings be viewed in conjunction with smaller but related projects that utilise more creative and participatory approaches (e.g., https://feelingathome.org.uk/; Kaley et al. 2022).

Despite the overall large sample size, participants from minority ethnic communities were underrepresented. Data suggests that proportionally less people from minority ethnic communities live in residential care or supported living compared with white people (NHS Digital 2021) which may have contributed to the issue. Responding to this limitation, a related project, Small Margins, focusing on people with intellectual disabilities and autistic people from minority ethnic communities followed on from this research (Leeson and Dunstan 2022). Lastly, the research occurred during the COVID‐19 pandemic which significantly disrupted people's routines and lifestyles. However, due to the increased time spent at home, the significance of home likely increased during this time, and it is reassuring that our findings align with previous research.

## 5 Conclusion

This paper explores what people with intellectual disabilities living in supported living and residential care like and dislike about their home. The findings indicate that space and place, people within the home and opportunities for control over daily life were appreciated. This complements existing research regarding how opportunities to shape and make a space one's own whilst paying attention to location are conducive to building a sense of belonging and creating a home. However, the presence of staff and housemates and the power yielded by others within and around the home, such as landlords, challenge normative constructs of home and pose an additional hurdle that people with intellectual disabilities must navigate in the creation of home. Policy needs to ensure greater availability and diversity of homely places to live, in neighbourhoods where people feel at home, and where people can make their home their own. People also need to be supported to choose who (if anyone) they live with, and for this to be able to change over time.

## Ethics Statement

I can confirm the study was conducted in adherence to ethical guidelines and received ethical approval from the HRA Social Care Research Ethics Committee ref.: 20/IEC08/0041.

## Conflicts of Interest

The authors declare no conflicts of interest.

## Data Availability

The data that support the findings of this study are available on request from the corresponding author. The data are not publicly available due to privacy or ethical restrictions.
